# Supplementary material for: A broadly protective human monoclonal antibody targeting the sialidase activity of influenza A and B virus neuraminidases
Source: Nat Commun. 2022 Nov 3;13:6602. doi: 10.1038/s41467-022-34521-0 (PMC9632566; doi:10.1038/s41467-022-34521-0)
Supplement: Supplementary file 1 — Supplementary Information [file 41467_2022_34521_MOESM1_ESM.pdf]

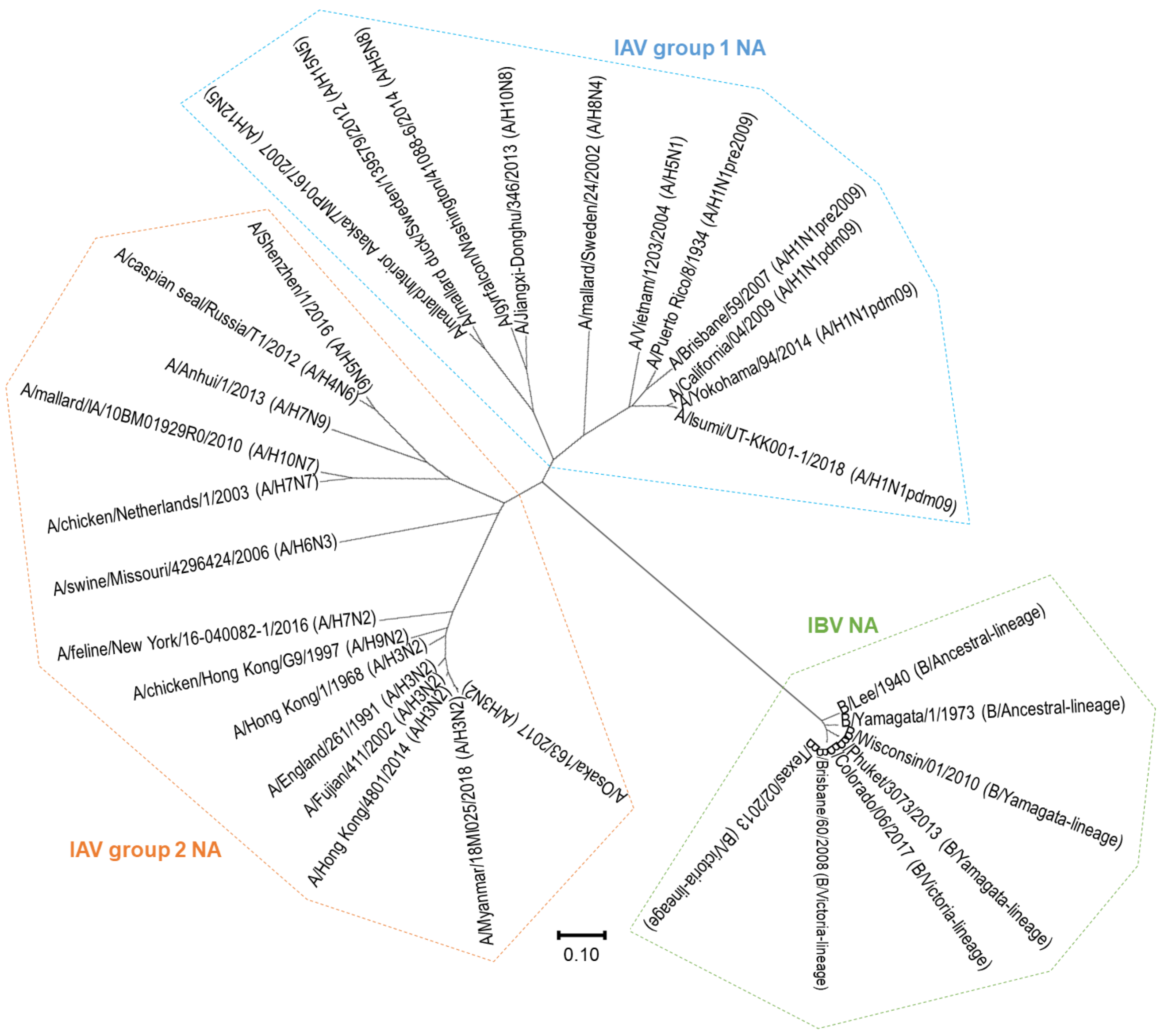

Supplementary Figure 1. Phylogenetic tree of influenza A and B virus NAs. The scale bar represents a 10% change in amino acids. The tree was built using amino acid sequences in MEGA11. Source data are provided as a Source Data file.

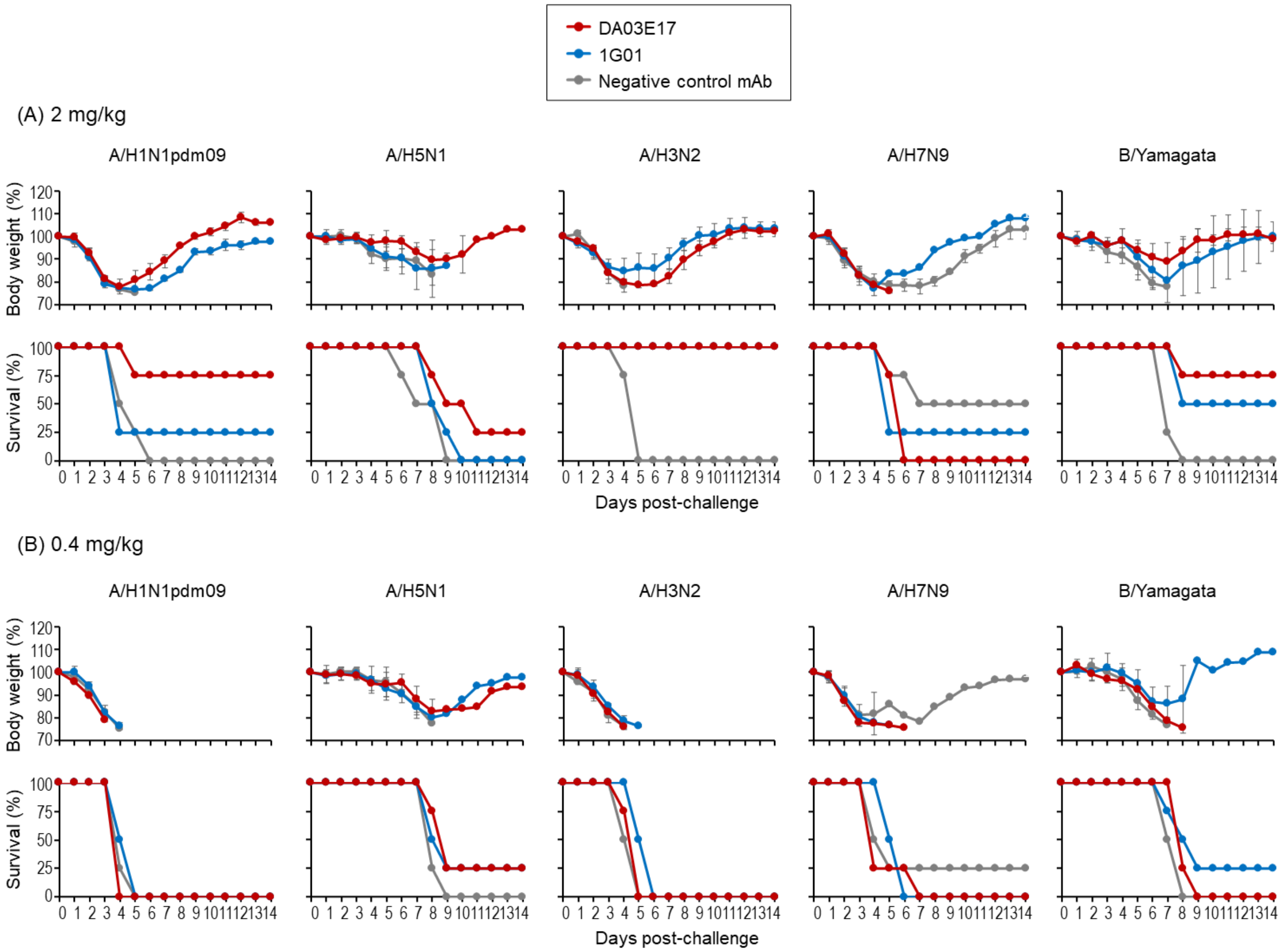

Supplementary Figure 2. In vivo protective efficacy of low-dose mAbs. Four mice per group were intraperitoneally inoculated with DA03E17 (red line), 1G01 (blue line), or the negative control mAb (grey line) at 2 mg/kg (A) or 0.4 mg/kg (B); 1430E3/9 and F3A19 were used as negative controls for IAVs and an IBV, respectively. One day later, the mice were challenged with 10 MLD50 of the indicated challenge viruses. Body weight changes and survival were monitored daily for 14 days. Body weight changes are shown as the mean ± SDs. Source data are provided as a Source Data file.

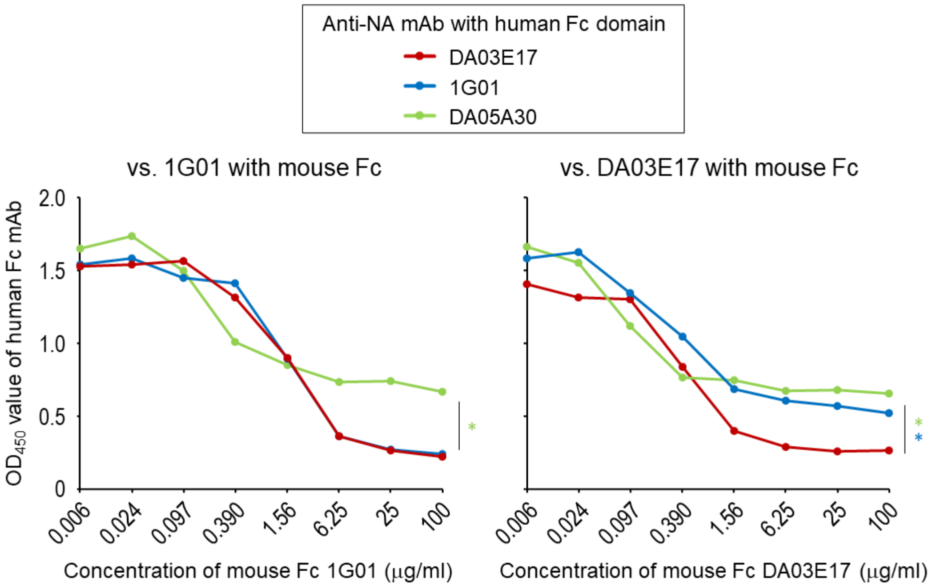

Supplementary Figure 3. Competitive binding between anti-NA human monoclonal antibodies and DA03E17 or 1G01 possessing mouse IgG2a. VP40-induced VLPs presenting A/H1N1pdm09-NA were used as the antigen. Serially diluted (100–0.006 µg/ml) mouse Fc 1G01 (left panel) or mouse Fc DA03E17 (right panel), whose Fc region was exchanged with the mouse Fc region, were incubated with the VLP to block their epitope on NA. Then, the indicated human monoclonal antibody (DA03E17, 1G01, or DA05A30) at concentrations adjusted to achieve an OD450 value of 1.5 was added. An HRP-conjugated goat anti-human IgG, Fcγ Fragment-specific antibody was used as the secondary antibody. The OD450 value of the human Fc antibody in the presence of each concentration of mouse Fc antibody was measured. *$P < 0.01$ (two-way ANOVA followed by Dunnett's tests). Source data are provided as a Source Data file.

Supplemental table 1. Genetic features of human mAbs against NA.

mAb	Heavy chain			Light chain		
	VH[b]	Mutation rate (%)[c]	CDR3[d]	VL[e]	Mutation rate (%)	CDR3
HP02A67	IGHV3-15*01	6.3	ITDTVNYSGGAY	IGLV6-57*04	3.5	QSYDGSDHWV
DA03E17	IGHV4-31*03	6.7	ARVDGSGNTDRYYFYGMDV	IGKV1-12*01	3.6	QQADGWEVWT
DA05A30	IGHV5-51*01	6.4	ARHPQPYGSGYYGEFDY	IGLV1-40*01	3.5	QSYDSSLSGSVV
1G01[a]	IGHV3-20*04	9.5	ARTSSWGDYTRGPEPKITWYFDL	IGKV1-9*01	6.0	QHLDSYPLFT

[a] 1G01 was used as a positive control.
[b] Variable gene for the heavy chain.
[c] The sequences of the VH or VL regions of each mAb were compared with their germline sequence.
[d] Complementarity determining region 3.
[e] Variable gene for the light chain.

Supplemental table 2. The minimal binding concentration (μg/ml) of mAbs to NA-displaying VLPs.

Subtype	Origin of NA tested	HP02A67	DA03E17	DA05A30	1G01	1430E3/9
A/H1N1 pre2009	A/Puerto Rico/8/34	>50	0.0488	12.5	>50	>50
	A/Brisbane/59/2007	12.5	<0.003	50	0.0122	>50
A/H1N1 pdm09	A/California/04/2009	12.5	<0.003	3.13	0.0122	>50
	A/Yokohama/94/2015	12.5	0.0488	12.5	0.195	>50
	A/Isumi/UT-KK001-1/2018	50	0.0488	12.5	0.0122	>50
A/H5N1	A/Vietnam/1203/2004	>50	12.5	>50	0.781	>50
A/H8N4	A/mallard/Sweden/24/2002	- [a]	0.78	-	<0.003	>50
A/H12N5	A/mallard/Interior Alaska/7MP0167/2007	-	0.012	-	0.78	>50
A/H15N5	A/mallard duck/Sweden/139579/2012	-	12.5	-	0.78	>50
A/H5N8	A/gyrfalcon/Washington/41088-6/2014	-	<0.003	-	<0.003	>50
A/H10N8	A/Jiangxi-Donghu/346/2013	-	3.1	-	0.012	>50
A/H3N2	A/Hong Kong/1/68	>50	0.0488	>50	<0.003	>50
	A/England/261/91	>50	3.13	>50	<0.003	>50
	A/Fujian/411/2002	>50	<0.003	>50	<0.003	>50
	A/Hong Kong/4801/2014	>50	<0.003	>50	<0.003	>50
	A/Osaka/163/2017	-	0.049	-	0.049	>50
	A/Myanmar/18MI025/2018	-	0.049	-	0.20	>50
A/H7N2	A/feline/New York/16-040082-1/2016	-	<0.003	-	<0.003	>50
A/H9N2	A/chicken/Hong Kong/G9/1997	-	0.049	-	0.049	>50
A/H6N3	A/swine/Missouri/4296424/2006	-	<0.003	-	<0.003	>50
A/H4N6	A/Caspian seal/Russia/T1/2012	-	0.049	-	0.78	>50

A/H5N6	A/Shenzhen/1/2016	-	0.012	-	0.012	>50
A/H7N7	A/chicken/Netherlands/1/2003	-	0.012	-	0.012	>50
A/H10N7	A/mallard/IA/10BM01929R0/2010	-	0.012	-	0.20	>50
A/H7N9	A/Anhui/1/2013	>50	0.781	>50	0.0122	>50
B/Ancestral-lineage	B/Lee/40	>50	50	>50	3.13	>50
	B/Yamagata/1/73	>50	3.13	>50	12.5	>50
B/Yamagata-lineage	B/Wisconsin/01/2010	>50	50	>50	50	>50
	B/Phuket/3073/2013	>50	<0.003	>50	0.781	>50
B/Victoria-lineage	B/Brisbane/60/2008	>50	3.13	>50	50	>50
	B/Texas/02/2013	>50	0.0488	>50	>50	>50
	B/Colorado/06/2017	>50	0.781	>50	0.781	>50

[a] Not tested.

Supplemental table 3. IC_{50} value (μg/ml) of neuraminidase inhibition activity against NA-VLPs.

Subtype	Origin of NA tested	HP02A67	DA03E17	DA05A30	1G01	1430E3/9
A/H1N1 pre2009	A/Puerto Rico/8/34	>50	1.08	>50	>50	>50
	A/Brisbane/59/2007	>50	0.0684	>50	0.0462	>50
A/H1N1 pdm09	A/California/04/2009	>50	0.00469	>50	0.0118	>50
	A/Yokohama/94/2015	>50	0.145	>50	2.81	>50
	A/Isumi/UT-KK001-1/2018	>50	1.82	>50	0.538	>50
A/H5N1	A/Vietnam/1203/2004	>50	0.00229	>50	0.00422	>50
A/H8N4	A/mallard/Sweden/24/2002	- [a]	>50	-	1.7	>50
A/H12N5	A/mallard/Interior Alaska/7MP0167/2007	-	7.2	-	>50	>50
A/H15N5	A/mallard duck/Sweden/139579/2012	-	42.8	-	>50	>50
A/H5N8	A/gyrfalcon/Washington/41088-6/2014	-	0.81	-	0.29	>50
A/H10N8	A/Jiangxi-Donghu/346/2013	-	0.16	-	<0.012	>50
A/H3N2	A/Hong Kong/1/68	>50	18.6	>50	0.376	>50
	A/England/261/91	>50	44.0	>50	0.245	>50
	A/Fujian/411/2002	>50	0.0889	>50	0.241	>50
	A/Hong Kong/4801/2014	>50	1.41	>50	0.375	>50
	A/Osaka/163/2017	-	30.3	-	4.2	>50
	A/Myanmar/18MI025/2018	-	47.1	-	7.2	>50
A/H7N2	A/feline/New York/16-040082-1/2016	-	0.03	-	0.022	>50
A/H9N2	A/chicken/Hong Kong/G9/1997	-	0.012	-	<0.012	>50
A/H6N3	A/swine/Missouri/4296424/2006	-	0.44	-	0.21	>50
A/H4N6	A/Caspian seal/Russia/T1/2012	-	0.029	-	0.073	>50

A/H5N6	A/Shenzhen/1/2016	-	0.74	-	3.5	>50
A/H7N7	A/chicken/Netherlands/1/2003	-	1.2	-	1.8	>50
A/H10N7	A/mallard/IA/10BM01929R0/2010	-	0.32	-	11.9	>50
A/H7N9	A/Anhui/1/2013	>50	28.7	>50	0.754	>50
B/Ancestral-lineage	B/Lee/40	>50	17.5	>50	0.118	>50
	B/Yamagata/1/73	>50	0.0389	>50	0.656	>50
B/Yamagata-lineage	B/Wisconsin/01/2010	>50	11.7	>50	40.3	>50
	B/Phuket/3073/2013	>50	0.522	>50	>50	>50
B/Victoria-lineage	B/Brisbane/60/2008	>50	2.64	>50	15.3	>50
	B/Texas/02/2013	>50	0.0756	>50	>50	>50
	B/Colorado/06/2017	>50	0.157	>50	0.0199	>50

[a] Not tested.

Supplemental table 4. IC_{50} value (μg/ml) of neutralization activity against influenza A or B virus.

Subtype	Virus isolate tested	HP02A67	DA03E17	DA05A30	1G01	1430E3/9	F3A19
A/H1N1 pre2009	A/Puerto Rico/8/34	>50	8.84	>50	>50	>50	-
	A/Brisbane/59/2007	>50	6.25	>50	4.96	>50	-
A/H1N1 pdm09	A/California/04/2009	>50	4.96	>50	1.56	>50	-
	A/Yokohama/94/2015	>50	6.25	>50	8.84	>50	-
	A/Isumi/UT-KK001-1/2018	>50	4.96	>50	4.42	>50	-
A/H5N1	A/Vietnam/1203/2004	>50	15.7	>50	0.984	>50	-
A/H3N2	A/Hong Kong/1/68	>50	19.8	>50	6.25	>50	-
	A/England/261/91	>50	>50	>50	4.42	>50	-
	A/Fujian/411/2002	>50	1.24	>50	1.10	>50	-
	A/Hong Kong/4801/2014	>50	8.84	>50	1.24	>50	-
A/H7N9	A/Anhui/1/2013	>50	3.94	>50	3.94	>50	-
B/Ancestral-lineage	B/Lee/40	>50	>50	>50	35.4	- [a]	>50
	B/Yamagata/1/73	>50	15.8	>50	17.7	-	>50
B/Yamagata-lineage	B/Wisconsin/01/2010	>50	>50	>50	>50	-	>50
	B/Phuket/3073/2013	>50	8.84	>50	>50	-	>50
B/Victoria-lineage	B/Brisbane/60/2008	>50	12.5	>50	>50	-	>50
	B/Texas/02/2013	>50	15.6	>50	>50	-	>50
	B/Colorado/06/2017	>50	9.92	>50	31.5	-	>50

[a] Not tested.

Supplemental table 5. Conservation rate of indicated amino acids in each subtype or lineage.

Amino acid	A/H1N1 pre2009	A/H1N1 pdm09	A/H5N1	A/H3N2	A/H7N9	B/Ancestral-lineage	B/Yamagata-lineage	B/Victoria-lineage
D151	98.9%	99.9%	100%	99.0%	100%	100%	100%	100%
T438	99.8%	99.8%	99.3%	100%	99.9%	-[a]	-	-
Total number of analyzed strains	3715	36411	436	57066	1266	16	13759	18176

[a] Threonine at position 438 is not shared by influenza B viruses.
