# Supplementary material for: A broadly protective human monoclonal antibody targeting the sialidase activity of influenza A and B virus neuraminidases
Source: Nat Commun. 2022 Nov 3;13:6602. doi: 10.1038/s41467-022-34521-0 (PMC9632566; doi:10.1038/s41467-022-34521-0)
Supplement: Supplementary file 3 — Reporting Summary [file 41467_2022_34521_MOESM3_ESM.pdf]

nature research

Corresponding author(s): Yoshihiro Kawaoka

Reporting Summary

Nature Research wishes to improve the reproducibility of the work that we publish. This form provides structure for consistency and transparency in reporting. For further information on Nature Research policies, see Authors & Referees and the Editorial Policy Checklist.

Statistical parameters

When statistical analyses are reported, confirm that the following items are present in the relevant location (e.g. figure legend, table legend, main text, or Methods section).

n/a	Confirmed	
☐	☒	The exact sample size (n) for each experimental group/condition, given as a discrete number and unit of measurement
☐	☒	An indication of whether measurements were taken from distinct samples or whether the same sample was measured repeatedly
☐	☒	The statistical test(s) used AND whether they are one- or two-sided<br>*Only common tests should be described solely by name; describe more complex techniques in the Methods section.*
☐	☒	A description of all covariates tested
☐	☒	A description of any assumptions or corrections, such as tests of normality and adjustment for multiple comparisons
☐	☒	A full description of the statistics including central tendency (e.g. means) or other basic estimates (e.g. regression coefficient) AND variation (e.g. standard deviation) or associated estimates of uncertainty (e.g. confidence intervals)
☐	☒	For null hypothesis testing, the test statistic (e.g. F, t, r) with confidence intervals, effect sizes, degrees of freedom and P value noted<br>*Give P values as exact values whenever suitable.*
☒	☐	For Bayesian analysis, information on the choice of priors and Markov chain Monte Carlo settings
☒	☐	For hierarchical and complex designs, identification of the appropriate level for tests and full reporting of outcomes
☒	☐	Estimates of effect sizes (e.g. Cohen's d, Pearson's r), indicating how they were calculated
☐	☒	Clearly defined error bars<br>*State explicitly what error bars represent (e.g. SD, SE, CI)*

Our web collection on statistics for biologists may be useful.

Software and code

Policy information about availability of computer code

Data collection	ELISA, ELLA, and competitive binding assay were performed by using Softmax Pro, v5.4.5 (Molecular Devices). NA-Star assay was performed with a microtiter plate reader (Turner BioSystems). The germline sequence was estimated in IgBlast software (https://www.ncbi.nlm.nih.gov/igblast/).
Data analysis	Statistical analysis was performed by using GraphPad Prism software v9.3.1. Structural analysis was performed by using the PyMOL molecular graphics system v2.5.2.

For manuscripts utilizing custom algorithms or software that are central to the research but not yet described in published literature, software must be made available to editors/reviewers upon request. We strongly encourage code deposition in a community repository (e.g. GitHub). See the Nature Research guidelines for submitting code & software for further information.

April 2018

Data

Policy information about availability of data

All manuscripts must include a data availability statement. This statement should provide the following information, where applicable:
- Accession codes, unique identifiers, or web links for publicly available datasets
- A list of figures that have associated raw data
- A description of any restrictions on data availability

All data analyzed during this study are included in this article. The raw data generated in this study are provided in the Source Data file. Antibody sequences have been deposited in GenBank [accession numbers: OP311729 (HP02A67 Heavy chain), OP311734 (HP02A67 Light chain), OP311730 (DA03E17 Heavy chain), OP311732 (DA03E17 Light chain), OP311731 (DA05A30 Heavy chain), and OP311733 (DA05A30 Light chain)]. The NA sequences of human isolates were collected from the GISAID database (https://www.gisaid.org/).

Field-specific reporting

Please select the best fit for your research. If you are not sure, read the appropriate sections before making your selection.

☒ Life sciences ☐ Behavioural & social sciences ☐ Ecological, evolutionary & environmental sciences

For a reference copy of the document with all sections, see nature.com/authors/policies/ReportingSummary-flat.pdf

Life sciences study design

All studies must disclose on these points even when the disclosure is negative.

Sample size	Sample sizes were similar to those generally employed in the field. All the experiments have been replicated at least twice.
Data exclusions	No data was excluded.
Replication	In vitro assays were performed in 2-3 independent. In vivo protection test was performed with four mice per experimental group. All attempts at replication were successful.
Randomization	Randomization was not necessary for the experiments. In the case of in vitro assays or in vivo challenge, randomization is not possible.
Blinding	Investigators had no information on the infected patients who provided PBMCs. For experiments other than those involving humans, blinding was not performed as it was not necessary because the experimental conditions were known to the researchers. All the data generated was analyzed with the same workflow. Data were analyzed using unbiased statistical tests.

Reporting for specific materials, systems and methods

Materials & experimental systems

n/a	Involved in the study
☒	☐ Unique biological materials
☐	☒ Antibodies
☐	☒ Eukaryotic cell lines
☒	☐ Palaeontology
☐	☒ Animals and other organisms
☐	☒ Human research participants

Methods

n/a	Involved in the study
☒	☐ ChIP-seq
☒	☐ Flow cytometry
☒	☐ MRI-based neuroimaging

Antibodies

Antibodies used	HRP-conjugated goat anti-human IgG, Fcγ Fragment-specific antibody (Code number: 109-035-098; Lot number: 149623) purchased from Jackson Immuno-Research (West Grove, PA, USA) was used as a secondary antibody in ELISA and competitive ELISA.<br>Human antibodies used in this study include: HP02A67 (this study), DA03E17 (this study), DA05A30 (this study), 1G01, 1430E3/9<br>All human antibodies identified in this study were made recombinantly by cloning antibody heavy and light chains into the mammalian expression vectors. Antibodies were produced in mammalian cells (Expi293 cells) by transient transfection of expression vectors and purified by protein A affinity chromatography.

Validation	HP02A67, DA03E17, and DA05A30 were validated in this study by sequencing, ELISA, and functional activity. The sequence, binding property, and functional activity of 1G01 or 1430E3/9 have been described in Stadlbauer et al. Science 2019, or Yasuhara et al. Nat Microbiol. 2019, respectively.<br>The goat anti-human Ab conjugated to HRP (Code number: 109-035-098; Lot number: 149623) purchased from Jackson Immuno-Research (West Grove, PA, USA) was validated in numerous previous scientific publications and by the manufacturer.

Eukaryotic cell lines

Policy information about cell lines

Cell line source(s)	Humanized Madin-Darby canine kidney (hCK) cells established in our lab (Takada et al. Nat Microbiol. 2019); 293T cells (ATCC); SPYMEG cells (MBL); Expi293F cells (Thermo Fisher Scientific)
Authentication	None of the cell lines used have been authenticated.
Mycoplasma contamination	All used cell stocks tested negative for mycoplasma.
Commonly misidentified lines (See ICLAC register)	No commonly misidentified cell lines were used.

Animals and other organisms

Policy information about studies involving animals; ARRIVE guidelines recommended for reporting animal research

Laboratory animals	Six-week-old female BALB/c or DBA2 mice (Japan SLC) were used in the study. The relative humidity was kept at 45 to 65%. Mouse rooms and cages were kept at a temperature range of 20 to 24°C, and were set at a 12:12 for the light:dark cycle. All experiments with mice were performed in accordance with the University of Tokyo's Regulations for Animal Care and Use and were approved by the Animal Experiment Committee of the Institute of Medical Science, the University of Tokyo.
Wild animals	No wild animals were used in the study.
Field-collected samples	No field collected samples were used in the study.

Human research participants

Policy information about studies involving human research participants

Population characteristics	PBMCs obtained from volunteers who were infected with the A/H1N1pdm09 virus in the 2015–2016 influenza season were used for hybridoma generation. The volunteers were recruited regardless of sex or age.
Recruitment	Posters were posted on bulletin board of Research Hospital and our institute. Subjects were faculty member, students, their families and others who got interested in this project. The investigators were not involved in recruitment of volunteers and have no knowledge of any volunteer information. No potential self-selection bias or any other bias in this study.
